# 4123 Characterization of Physical Restraint and Sedative Use for Treatment of Agitation in the Emergency Department

**DOI:** 10.1017/cts.2020.374

**Published:** 2020-07-29

**Authors:** Ambrose H Wong, Lauren Crispino, John Parker, Caitlin McVaney, Alana Rosenberg, Jessica Ray, Travis Whitfill, Joanne Iennaco, Steven Bernstein

**Affiliations:** 1 Yale School of Medicine

## Abstract

OBJECTIVES/GOALS: Agitation has high prevalence in the emergency department (ED), but limited evidence exists regarding clinical decisions to use sedatives and physical restraint. We examined clinical factors and agitation attributes impacting thresholds for sedative and restraint use in the emergency setting. METHODS/STUDY POPULATION: We conducted a prospective cohort study of adult patients (^3^18 yo) with acute or escalating agitation during their ED visit at an urban tertiary care referral center. Consecutive patients requiring security presence or scoring >1 on an agitation scale were enrolled during randomized 8-h blocks. We recorded patient characteristics, staff/team factors, and environmental/systems data as well as scores on 3 validated agitation scales: Agitated Behavior Scale, Overt Aggression Scale, and Severity Scale. We performed descriptive analyses, bivariable analyses, and logistic regression modeling of factors with relation to sedative/restraint use. We observed 95 agitation events on unique patients over 2 months. RESULTS/ANTICIPATED RESULTS: Median age was 42, and 62.1% were male. Most frequent chief complaints were alcohol/drug use (37.9%) and psychiatric (23.2%). Majority of events (73.7%) were associated with sedative/restraint use. Factors related to treatment course or staff interactions were the primary reasons for agitation in 56.8% of events. A logistic regression model found no association between demographics and odds of sedative/restraint use. Overt Aggression Scale scores were associated with significantly higher odds of sedative use (AOR 1.62 [1.13–2.32]), while Severity Scale scores had significantly higher odds of restraint use (AOR 1.39 [1.12–1.73]) but significantly lower odds of sedative use (AOR 0.79 [0.64–0.98]). DISCUSSION/SIGNIFICANCE OF IMPACT: External factors may be important targets for behavioral techniques in ED agitation management. Further study of the Severity Scale may allow for earlier detection of agitation and identify causal links between agitation severity and use of sedatives and restraints.

